# Depression and anxiety during and after episodes of COVID-19 in the community

**DOI:** 10.1038/s41598-023-33642-w

**Published:** 2023-05-22

**Authors:** Caterina Alacevich, Inna Thalmann, Catia Nicodemo, Simon de Lusignan, Stavros Petrou

**Affiliations:** 1 Nuffield Department of Primary Care Health Sciences, University of Oxford, Oxford, UK; 2 Health Outcomes and Biomedical Informatics, University of Florida, Gainesville, USA; 3 Nuffield Department of Population Health, University of Oxford, Oxford, UK; 4 University of Verona, Verona, Italy

**Keywords:** Risk factors, Signs and symptoms, Infectious diseases, Anxiety, Depression, Public health

## Abstract

Understanding the connection between physical and mental health with evidence-based research is important to inform and support targeted screening and early treatment. The objective of this study was to document the co-occurrence of physical and mental health conditions during and after the experience of symptomatic SARS-CoV-2 illness episodes. Drawing from a national symptoms' surveillance survey conducted in the UK in 2020, this study shows that individuals with symptomatic forms of SARS-CoV-2 (identified by anosmia with either fever, breathlessness or cough) presented significantly higher odds of experiencing moderate and severe anxiety (2.41, CI 2.01–2.90) and depression (3.64, CI 3.06–4.32). Respondents who recovered from physical SARS-CoV-2 symptoms also experienced higher odds of anxiety and depression in comparison to respondents who never experienced symptoms. The findings are robust to alternative estimation models that compare individuals with the same socioeconomic and demographic characteristics and who experienced the same local and contextual factors such as mobility and social restrictions. The findings have important implications for the screening and detection of mental health disorders in primary care settings. They also suggest the need to design and test interventions to address mental health during and after physical illness episodes.

## Introduction

Identifying conditions associated with anxiety and depression is important to inform and improve targeted screening and early treatment. During the COVID-19 pandemic, the global prevalence rates of depression and anxiety ranged between 21.3–24% and 31.9–33.7%, respectively, according to two meta-analyses^1,2^, significantly higher than the levels reported in pre-COVID-19 times (2–6% and 2.5–7% in 2017)^3^. A number of studies have sought to examine the factors associated with this deterioration in mental health status during the pandemic across different countries and found that individuals’ economic losses, social isolation, health risks and sociodemographic factors (i.e., sex and ethnicity) were significantly associated with an increase in the levels of depression and anxiety^4–10^.

However, there is less evidence of the relationship between the experience of common COVID-19 symptoms, especially without hospitalization, and mental health. A limited number of studies examined the association between self-reported symptomatic COVID-19 and mental health status and reported a clinically significant deterioration in mental health among infected individuals compared to those without a COVID-19 self-report in the United Kingdom^11^. Similar findings were observed in countries outside the United Kingdom, such as the United States and Northern European countries, showing that neurological and psychiatric morbidity were substantial within six months after a COVID-19 diagnosis^12,13^. In particular, patients hospitalized or bedridden for more than seven days due to COVID-19 had a higher hazard of being diagnosed with a mood, anxiety or psychiatric disorder and experiencing symptoms of anxiety and depression^12,13^. Should health care systems promote mental health prevention and treatment for patients that present SARS-CoV-2 symptoms? Should healthcare providers screen patients that recovered from physical symptoms for depression and anxiety? Answers to these timely questions require evidence-based informed discussions.

The aim of this study was to examine the relationship between symptomatic COVID-19 illness states and the likelihood of experiencing anxiety and depression, during and after illness episodes. It contributes to the literature by comparing anxiety and depression in respondents who reported experiencing symptomatic SARS-CoV-2 illness episodes (anosmia with breathlessness, high fever, or a new continuous cough) and having recovered from past COVID-19-related physical health symptoms to those who did not report any experience of COVID-19 physical symptoms during or before survey participation. We hypothesised that individuals who were experiencing COVID-19 symptoms would have significantly higher odds of experiencing anxiety and depression compared to those who did not have any COVID-19 physical symptoms. We also expected to find a positive association between physical and mental health conditions among individuals who recovered from COVID-19-related physical symptoms. The study addresses these hypotheses by drawing from a UK national Symptoms' Surveillance Survey and using validated screening instruments for anxiety and depression, namely the Generalised Anxiety Disorder (GAD-7) and Patient Health Questionnaire (PHQ-9)^14,15^.

Existing studies suggest that similar infections and viral conditions increased the risk of neurological or psychiatric morbidity sequelae^13,16–18^. As the literature on COVID-19 illness episodes and mental health is mostly based on small samples, hospitalised patients, qualitative research studies^17–20^, and formal diagnostic cases^13,21^, further evidence can improve our understanding.

Mental health conditions impose significant direct and indirect costs and burdens on those who experience them and their caregivers, with significant consequences for private and public health budgets^22–25^. Mental conditions are also associated with lower adherence to medication, treatment, and recommended healthy behaviours^26^. The worldwide number of Disability-Adjusted Life Years lost due to mental disorders had already reached 125.3 million in 2019 before the pandemic^27^. Understanding the relationship between physical and mental health can be crucial for defining current public health priorities, informing health service planning and screening interventions, and tackling the mental health legacies of the pandemic.

## Data and methods

This observational study uses survey data from a national digital symptoms' surveillance survey of the UK Royal College of General Practitioner, the University of Oxford, and EMIS Health. The survey collected 16,711 cross-sectional responses between April and December 2020 through Patient Access, a digital primary health care service tool. The COVID-19 Symptom Surveillance tool covered the underpinning research infrastructure and governance and approval and consent procedures for voluntary participation as articulated in EMIS Health’s privacy policy. The survey obtained informed consent from all participants and/or their legal guardian(s) if aged 16–18 years. EMIS Health processes personal and sensitive data under the legal basis of medical research or public interest. Medical research to answer legitimate research questions in the public interest is justified under schedule 1, sections 2–4 of the Data Protection Act 2018 and in the presence of appropriate data subject safeguards. The legal basis for EMIS’s processing of data is consent or approval for exemption under Section 251 of the NHS Act 2006. The data reported in this study were fully anonymized by EMIS Health and the research team were given access to a dataset stripped of all personal identifiers. As a result, the study was not subject to General Data Protection Regulation (GDPR) requirements or ethics review.

The data for this study included basic demographic characteristics and information on cohabitation, employment status, three-digit postcodes, comorbidities, smoking behaviour, validated screening modules to assess depression and anxiety, and lists of risky health conditions and current or past symptoms since the pandemic outbreak. To represent the demographic structure of the UK population, we computed and applied probability weights based on age-gender cell counts^28^ and additionally performed sensitivity analyses without weights. We classified respondents into three categories by identifying those who had COVID-19 viral symptoms, recovered from physical symptoms, or did not experience them using their reported experienced symptoms, either at survey completion or in any significant illness episode experienced before the survey. Following the literature^29,30^, we identified COVID-19 symptomatic episodes by selecting anosmia in combination with either high fever, a new continuous cough, or breathlessness. Respondents who reported that they did not experience any COVID-19 illness episode and did not report any other symptoms during or before the survey were defined as “never ill”. Respondents who recovered from the defining COVID-19 symptoms by the time they completed the survey were included in the “past illness” category. Respondents that experienced other symptoms but none of the above were not included in the study, neither as ill with COVID-19-related symptoms nor as “never ill”. We excluded 27 observations that reported anosmia but no cough/fever/breathlessness and 211 without anosmia from the original sample. The results (available upon request) are robust to their re-inclusion.

To measure anxiety, we utilized the GAD-7 module^14^, where a final score (1–21) aggregates seven 4-point Likert scale answers about symptoms manifestations over the previous two weeks. The threshold for moderate and severe anxiety (binary, ≥ 10) has 89% sensitivity and 82% specificity^14^. Depression screening relied on the PHQ-9 module of the PRIME-MD instrument^15^, which aggregates nine 4-point Likert scale answers into a single score (0–27). The binary threshold (≥ 10) for major and severe depression has 88% sensitivity and 88% specificity^15^. We assessed binary thresholds and overall scores. Appendix B describes the content of the GAD-7 and PHQ-9 questionnaire modules. The GAD-7 and PHQ-9 are popular screening tools for anxiety and depression, respectively, because they are brief, easy to use, and have been validated for use in a variety of settings. Both measures have been shown to be reliable and valid for use in clinical and research settings, and their brevity makes them easy to administer in busy clinical environments^31–33^.

### Econometric model

We estimated multivariable logistic regressions for binary outcomes based on the following model:

1 $$Logit\left( p \right) = \alpha_{i} + \beta COVIDSymptoms_{i} + \gamma Covariates_{i} + \tau + \theta Lockdown_{i,t} + \eta Region_{i} + \varepsilon_{i}$$

where $$p = P(Y_{i} = 1)$$ and $$Y_{i}$$ indicates, alternatively, the binary indicator for moderate/severe anxiety or depression. For the GAD7 anxiety score and the PHQ9 depression score outcomes, we estimated multivariable OLS regressions. The explanatory variable ($$COVIDSymptoms_{i} )$$ is the vector of COVID-19 illness states (current, recovered, or none—baseline category). We included an extensive set of individual fixed effects to compare respondents with the same demographic, socioeconomic, and health characteristics, and we included region and time ($$\tau$$) fixed effects. In addition to the individual-level characteristics, described in Table 1, covariates included also a category identifying missing data, where applicable, and an indicator for lockdowns and local mobility restrictions due to the pandemic. For sensitivity analyses, we repeated the estimations by (i) removing survey weights, (ii) excluding all regressors except COVID-19 illness status, (iii) including month fixed effects while excluding lockdown, and (iv) excluding observations with missing covariates.

## Results

Table 1 reports the descriptive statistics of the study population, including gender, age group, ethnic group, comorbidity (see Appendix A for details), highly risky health conditions (see Appendix A), employment status, annual household income, cohabitation, smoking behaviour, national/local lockdowns or local mobility restrictions^34,35^, location (region) of residency, and month of survey completion. 17.5% of the 16,771 respondents reported SARS-CoV-2 symptoms. 57.8% did not experience symptoms after the pandemic outbreak, and 24.7% recovered from a symptomatic episode. 23.9% and 30.2% reported moderate/severe anxiety and depression, respectively.

**Table 1:** Descriptive statistics: characteristics of the study population.

Variable	N	%		N	%
*COVID-19 illness status*			*Comorbidity*		
Never ill	10,208	57.85	No comorbidity	11,115	62.99
Current illness symptoms	3087	17.49	Has comorbidity	6209	35.19
Past illness symptoms	4351	24.66	Missing	322	1.83
*Anxiety*			*Risky health condition*		
Moderate-severe	2947	23.90	No risky condition	14,357	81.36
*Depression*			Risky condition	1782	10.10
Moderate-severe	3957	30.20	Missing	1508	8.54
*Age*			*Smoking behavior*		
Age 16–34	5212	29.54	Smoker	2069	11.72
Age 35–49	4173	23.65	Non-smoker	10,281	58.26
Age 50–64	4179	23.68	Past smoker	5297	30.02
Age 65 +	4082	23.13	*Mobility restrictions*		
Age missing	0	0	No restrictions	5066	28.71
*Gender*			Lockdown or Tier 3/4	12,580	71.29
Male	7109	40.29	*Region*		
Female	10,537	59.71	Scotland	712	4.04
Gender missing	0	0	Northern Ireland	193	1.09
*Ethnicity*			North East	822	4.66
White	12,187	69.06	North West	3295	18.67
Other Ethnic Group	1207	6.84	East Midlands	1171	6.64
Missing	4253	24.10	West Midlands	2159	12.23
*Employment status*			Wales	283	1.60
Not employed	2304	13.06	South West	1334	7.56
Self-employed	1089	6.17	South East	3670	20.80
Employed part-time	1915	10.85	Greater London	3295	18.68
Employed full-time	7447	42.20	Missing region	712	4.03
Retired	4408	24.98	*Month*		
Student–not employed	483	2.74	April	3632	20.58
*Annual income (GBP)*			May	8233	46.66
Less than 5200	125	0.71	June	214	1.21
5200–less than 18,200	656	3.72	July	90	0.51
18,200–less than 31,200	1118	6.34	August	33	0.18
31,200–less than 52,000	1253	7.10	September	524	2.97
52,000–less than 100,000	1101	6.24	October	4336	24.57
100,000 or more	361	2.05	November	360	2.04
Missing	13,031	73.85	December	224	1.27
*Co-habiting*					
Yes	14,756	83.62			
No	2890	16.38			

Descriptive statistics of the UK COVID-19 symptoms tracker survey (April–December 2020). Sample size: 16,771. Observations are weighted using estimated probability weights at the age-gender level. GAD-7 mean score is 5.98. PHQ-9 mean score is 7.17.

**Figure 1:**
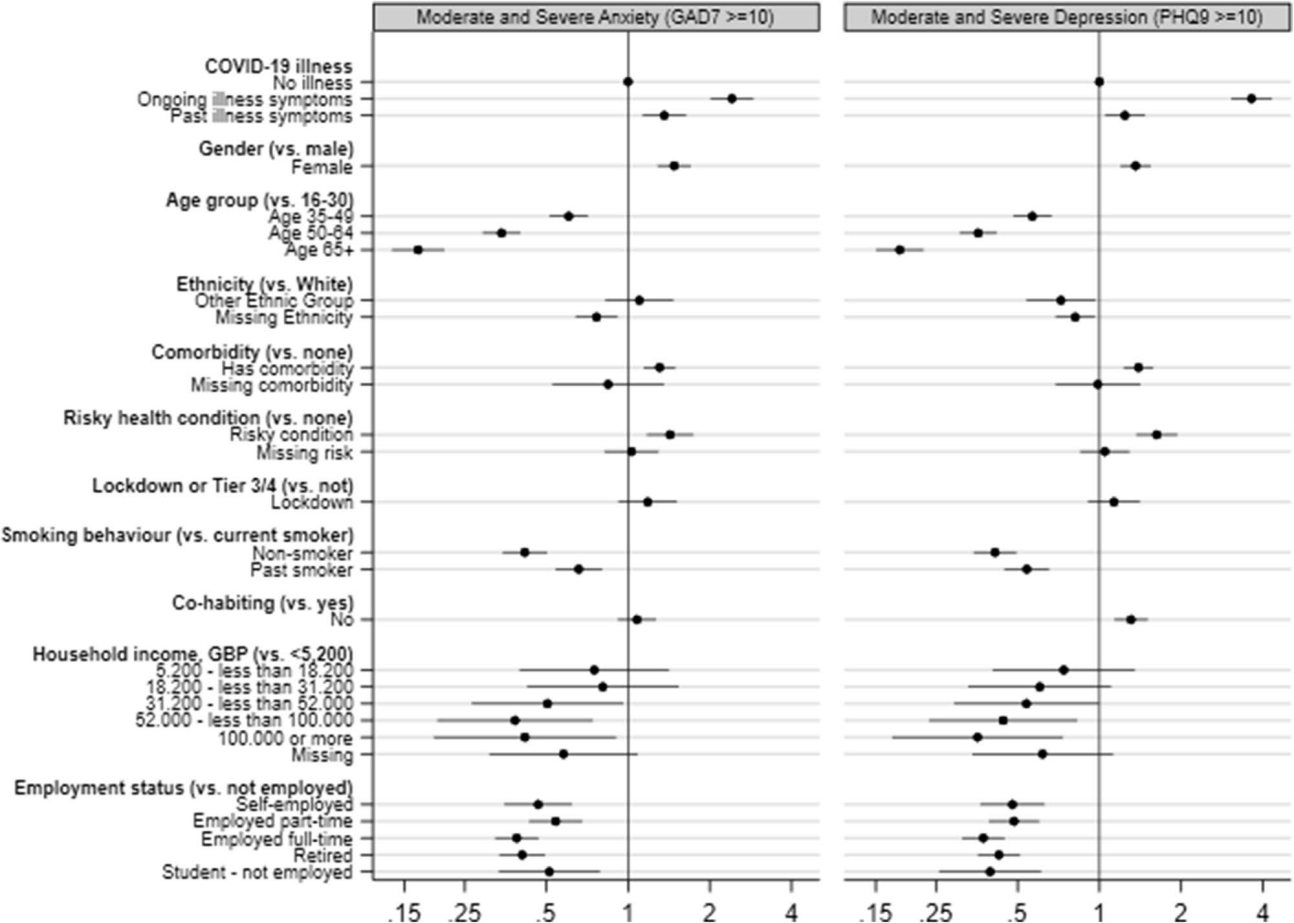
COVID-19 symptomatic episodes and mental health (adjusted odds ratios, logistic regression). Notes. Authors’ estimations from the UK COVID-19 symptoms tracker survey (April–December 2020). Adjusted ORs and 95% CI. X-axis on log scale. Sample size: 16,771. “Ongoing illness” identifies respondents with COVID-19 symptoms; “Past illness” identifies respondents that recovered from symptomatic COVID-19. Other covariates: region fixed effects and a constant. Outcomes (binary) are moderate and severe anxiety or depression, based on GAD-7 and PHQ-9 metrics. Regressions include age-gender-specific probability weights. Standard errors are heteroskedasticity-robust.

Notes. Authors’ estimations from the COVID-19 symptoms tracker survey (April-December 2020). Adjusted ORs and 95% CI. X-axis on log scale. Sample size: 16,771. “Current illness” identifies respondents with COVID-19 symptoms; "Past illness" identifies respondents that recovered from symptomatic COVID-19. Other covariates: region FE and a constant. Outcomes (binary) are moderate and severe anxiety or depression, based on GAD-7 and PHQ-9 metrics. Estimates include age-gender-specific probability weights. SEs are heteroskedasticity-robust.

Figure 1 (see also Table 2, column 1) displays the adjusted odds ratios (ORs) with 95% confidence intervals (CI) for moderate and severe anxiety or depression by symptomatic state and other fixed effects. All else equal, the odds ratios of moderate/severe anxiety and depression were, respectively, 2.41 (95% CI 2.01–2.90) and 3.64 (CI 3.06–4.32) amongst respondents with symptoms. Also, past symptomatic illness experiences were significantly associated with higher anxiety (OR 1.36, CI 1.13–1.63) and depression (OR 1.24, CI 1.05–1.47), at the 1% level (5% for anxiety, past illness).

Figure 1 also reports the ORs for the additional covariates. All else equal, depression and anxiety ORs were higher for females, < 30 years old (versus all other age groups), comorbidity or risky conditions, current and past smokers, living alone (for depression), unemployed (vs all other employment categories), white (vs missing ethnicity), and the lowest income group (vs 52,000 + GBPs).

### Robustness checks

Table 2 reports the results obtained from different sensitivity analyses (logistic regression in columns 1–5) and the results of OLS regressions for the continuous GAD-7 and PHQ-9 scores. Column 1 reports the odds ratios from the main model, for comparison (see Fig. 1). Col. 2 reports the results of the same regression without probability weights. Col. 3 excludes all covariates but the main explanatory variable, Col. 4 includes month-specific dummy variables while excluding mobility and social restriction measure fixed effects, and Col. 5 excludes all observations for which a covariate is missing. The results are consistent with the main estimations, confirming their robustness. Column 6 reports the results of OLS estimations for continuous anxiety and depression GAD7 and PHQ9 scores. The results show a + 2.7-points increase in GAD7 anxiety score (+ 0.46 SD, col. 6, Panel A) and a + 4.5-points increase in PHQ9 depression score (+ 0.7 SDs, col. 6, Panel B) among respondents with physical illness symptoms, significant at the 1% level. Participants who recovered from past COVID-19-related symptomatic illness displayed a + 0.13 SD-higher anxiety score and + 0.14 SD-higher depression score (1% level). Additional OLS (linear probability model) estimations showed that the probability of experiencing moderate/severe anxiety and depression was 16 and 27 percentage points (pp) higher among respondents with co-occurring physical symptoms and 4.9 and 3.8 pp higher among those who recovered than the “never ill” respondents (full results available upon request).

**Table 2:** COVID-19 illness state and anxiety and depression: sensitivity analysis.

	(1)	(2)	(3)	(4)	(5)	(6)
Panel A	Outcome: moderate and severe anxiety (GAD-7 ≥ 10) (logistic regressions)					GAD-7 score (OLS)
Reference: No illness						
Ongoing illness (symptoms)	2.413***	2.322***	2.889***	2.418***	1.924***	2.703***
	(0.225)	(0.159)	(0.205)	(0.225)	(0.352)	(0.220)
Past illness (symptoms)	1.359***	1.173**	1.449***	1.360***	1.773***	0.751***
	(0.128)	(0.077)	(0.105)	(0.128)	(0.338)	(0.194)
N	16,771	16,771	16,771	16,771	4699	16,771
Mean of dep. var	0.239	0.176	0.239	0.239	0.181	5.983
Std dev of dep. var	0.427	0.381	0.427	0.427	0.385	5.861

Panel B	Outcome: moderate and severe depression (PHQ-9≥10) (logistic regressions)					PHQ-9 score (OLS)
Reference: No illness						
Ongoing illness (symptoms)	3.636***	4.069***	4.157***	3.707***	3.434***	4.511***
	(0.320)	(0.262)	(0.279)	(0.326)	(0.575)	(0.261)
Past illness (symptoms)	1.243**	1.265***	1.378***	1.246**	1.486**	0.942***
	(0.108)	(0.077)	(0.092)	(0.108)	(0.270)	(0.222)
N	16,771	16,771	16,771	16,771	4699	16,771
Mean of dep. var	0.302	0.236	0.302	0.302	0.231	7.168
Std dev of dep. var	0.459	0.425	0.459	0.459	0.421	6.635

Authors’ estimations from the UK COVID-19 symptoms tracker survey (April-December 2020). “Ongoing illness (symptoms)” identifies respondents with COVID-19 compatible symptoms (anosmia and either cough, high fever, or breathlessness (= 1) versus those with no illness episode (= 0)). Columns 1 and 2 report the odds ratios from multivariable logit regressions. Col. 1 is the reference model, which includes weights computed from population estimates by gender and age. Additional covariates: gender, age group, ethnicity, comorbidity, highly risky health condition, smoking habit, cohabitation, household income, employment status, lockdown or Tier 3–4, and region fixed effects, with missing categories. Col. 2 does not use probability weights, Col. 3 excludes all covariates but the main explanatory variable, Col. 4 includes month-specific dummy variables while excluding mobility and social restriction measures, Col. 5 excludes all observations for which a covariate is missing. Col. 6 reports results of OLS regressions with the full set of covariates. Parentheses report heteroskedasticity-robust standard errors. Statistical significance levels: 10 (*), 5 (**), 1 (***) percent.

## Discussion and conclusion

This study documents that common physical SARS-CoV-2 health symptoms of anosmia and high fever, new continuous cough, or breathlessness were significantly associated with experiences of moderate and severe anxiety and depression during physical symptomatic COVID-19 episodes as well as after recovery. Similar findings were observed in a nationally representative longitudinal study of UK households investigating the relationship between probable COVID-19 symptoms and psychological distress measured via the General Health Questionnaire (GHQ-12), showing increased levels of clinically significant psychological distress up to seven months after probable COVID-19, compared with individuals with no likely infection^36^. Specifically, individuals experiencing symptoms had, respectively, 39% and 47% higher odds of experiencing psychological distress at months one and seven following the probable infection compared to individuals without probable infection (month 1: odds ratio (OR) 1.39 (95% CI: 1.10–1.76); month 7: OR 1.47 (95% CI: 1.04–2.07)^36^. These findings were further substantiated in a large study analysing data from eleven UK longitudinal studies, observing a significant longitudinal association between self-reported COVID-19 and deterioration in mental health and life satisfaction using various measures compared to individuals without COVID-19 based on serology and self-report^11^. However, no association with mental health outcomes was observed among individuals who had positive serology but did not self-report COVID-19^11^. These observations are consistent with findings from countries outside the United Kingdom. A study of over 236,379 patients with a COVID-19 diagnosis in the US showed that neurological and psychiatric morbidity in the six months after the infection were still substantial, with risks being greatest, but not limited to, patients with severe COVID-19^12^. The estimated incidence of a neurological or psychiatric diagnosis within six months after COVID-19 diagnosis was 33.6% (95% CI 33.2%-34.1%) and 46.4% (95% CI 44.8%-48.1%) among those admitted to a hospital intensive therapy unit. Compared to non-hospitalized individuals infected with influenza, those diagnosed with COVID-19 without hospitalization had a 49% greater hazard of being diagnosed with a mood, anxiety or psychotic disorder (hazard ratio (HR) 1.49 (95% CI 1.45–1.54)). Those hospitalized for COVID-19 had a 23% greater hazard of a mood, anxiety or psychotic disorder diagnosis than those infected but not hospitalized for COVID-19 (HR 1.23 (95% CI 1.18–1.28)^12^. Similar mental health morbidity trajectories were observed across five Nordic countries, where severe acute COVID-19 illness was significantly associated with long-term mental morbidity among recovering individuals^13^. For instance, COVID-19 patients who were bedridden for more than seven days were persistently at higher risk of symptoms of depression (prevalence ratio (PR) 1.61 (95% CI 1.27–2.05)) and anxiety (PR 1.43 (95% CI 1.26–1.63)) than those without a diagnosis^13^.

The inclusion of an extensive set of individual fixed effects allowed us to isolate the association between SARS-CoV-2-related physical health symptoms and mental health from other general stressors and mediators of mental health conditions that were previously reported in studies examining associations between individual factors and mental health status during the pandemic, such as socioeconomic, demographic, location, and contextual factors, including mobility restrictions^1,18,37^. Nonetheless, additional factors that the survey did not capture may have contributed to determining COVID-19 exposure and mental health outcomes. The results of this study represent a description of co-occurring physical and mental health conditions and should not be interpreted in a causal way. These descriptive findings are relevant because they justify future research and larger/representative causal inference studies aimed at screening and supporting mental health during and after similar symptomatic illness experiences.

Due to limited availability of testing during the first periods of the pandemic in the UK, this study identified COVID-19-related symptomatic status based on reported symptoms of anosmia in combination with either high fever, breathlessness, or a new and continuous cough. This included respondents with other co-occurring symptoms. However, this classification may be restrictive and the results of this study do not generalize to individuals who experienced other symptoms and none of the above. The interpretation of the results should also consider that respondents classified as “never ill” may also have included those who experienced asymptomatic forms of COVID-19 and those who were classified as currently or previously ill may have also experienced multiple illness episodes. Furthermore, viral infections may trigger symptoms, such as sleep issues, appetite loss, and fatigue, which also contribute to mental health conditions. This study does not identify whether anxiety and depression have viral origins or speak to broader co-occurring quality of life considerations.

The study was conducted in 2020, during periods of frequent national lockdowns, limited understanding of the virus and absence of treatments and vaccines, factors which may have contributed to more severe deteriorations in mental health compared to the years after lockdown restrictions were eased and vaccines and treatments became available. The interpretation of the results may thus not be generalizable to different periods. However, Covid-19 infections continued to impose a substantial disease burden due to higher risk of severe illness and death and significantly longer incubation periods compared to influenza-like illnesses (ILIs), and were therefore associated with a lower health-related quality of life, including psychosocial health status, compared to ILIs^38^.

Another possible limitation is that voluntary participation could result in “collider bias”^39^ if survey response depended on factors that correlate with the outcomes and for which the survey did not collect and provide information (e.g.,^40^). Nevertheless, targeted voluntary recruitment and snowball sampling strategies are extensively employed in large scale surveys (see, e.g.,^37,41,42^). A source of validation for our results is that they resemble those based on *perceived* self-reported COVID-19 infection in Shevlin et al. (2020), which draw from a representative sample of the UK population^8^. Their estimates, based on a combined indicator of anxiety and depression ranged between 1.14–4.11, overlapping with our results (ORs of 2.41 and 3.63 for anxiety and depression among currently ill respondents). Further, when we computed the prevalence of anxiety and depression for the overall sample, we found results similar to other studies that were based on the UK general population from the same period (e.g.,^8,43^). These similarities reinforce the validity of our findings.

Estimates based on the UK population showed that mental health issues increased by 13.5 pp between 2017–2019 and April 2020 in the UK^43^. Our findings suggest that, during 2020, an even larger gap emerged between the surveyed population who experienced COVID-19 symptomatic illness and those who did not present symptoms (by 16 and 27 pp for anxiety and depression). This study documented also higher depression and anxiety among respondents who recovered from the COVID-19-specific physical health symptoms. The findings from our study suggest that what emerges as a mental health crisis from general population statistics might be the top of a wider hidden 'iceberg'^44^.

Identifying health factors that co-occur with adverse mental health conditions is a first crucial step to inform priorities for health and social care policy and planning. Understanding the relationship between physical viral-like symptoms and conditions such as anxiety and depression can improve the personalization of behavioural health screening and treatments and help to address the mental health legacies of the current pandemic. The present findings have important implications for the screening and detection of mental health disorders. Earlier detection and intervention for mental health conditions can improve patient outcomes and reduce healthcare costs. Building on the results of this study, future research could investigate mental health sequelae and interventions in post-COVID episodes in different populations and with varying time horizons. Other venues for research include a focus on groups that were most severely hit by COVID-19 and that also present social and demographic vulnerabilities and limited access to mental health care.

## Supplementary Information

Supplementary Information.

## Data Availability

The data that support the findings of this study are available from EMIS Health in collaboration with the University of Oxford and the UK Royal College of General Practitioners but restrictions apply to the availability of these data, which were used under license agreement for the current study, and so are not publicly available. Anonymized data are however available from the authors upon reasonable request and with permission of EMIS Health, the University of Oxford, and the UK Royal College of General Practitioners.
