# Supplementary material for: Depression and anxiety during and after episodes of COVID-19 in the community
Source: Sci Rep. 2023 May 22;13:8257. doi: 10.1038/s41598-023-33642-w (PMC10201488; doi:10.1038/s41598-023-33642-w)
Supplement: Supplementary file 1 — Supplementary Information. [file 41598_2023_33642_MOESM1_ESM.docx]

**Appendix to the manuscript**

**“Depression and Anxiety during and after episodes of COVID-19 in the community”**

Caterina Alacevich, Inna Thalmann, Catia Nicodemo, Simon de Lusignan, Stavros Petrou

**Description of the mental health screening instruments content and of the baseline health risk questions**

GAD-7 symptoms include (1) Feeling nervous, anxious or on edge; (2) Not being able to stop or control worrying; (3) Worrying too much about different things; (4) Having trouble relaxing; (5) Being so restless that it is hard to sit still; (6) Becoming easily annoyed or irritable; and (7) Feeling afraid as if something terrible might happen. For each question, respondents selected between "not at all", "several days", "more than half the days", and "nearly every day", corresponding to a score between 0 and 3. (Spitzer et al., 2006)

The PHQ9 items include: "(1) Little interest or pleasure in doing things, (2) Feeling down, depressed, or hopeless, (3) Trouble falling or staying asleep, or sleeping too much, (4) Feeling tired or having little energy, (5) Poor appetite or overeating (6) Feeling bad about yourself - or that you are a failure or have let yourself or your family down, (7) Trouble concentrating on things, such as reading the newspaper or watching television, (8) Moving or speaking so slowly that other people could have noticed? Or the opposite, [...] fidgety or restless [...], (9) Thoughts that you would be better off dead, or of hurting yourself in some way". (Kroenke et al., 2001)

Comorbidity: reporting any chronic health condition between "Lung disease such as asthma or COPD, Heart disease, Chronic kidney disease, Liver disease such as hepatitis, Nervous system conditions such as Parkinson's or MS, Diabetes, Problems with spleen, weakened immune system such as being on long term steroid tablets or having AIDS, Seriously overweight, i.e., BMI ≥40").

Highly Risky Health Conditions: any among organ transplant, pregnancy with heart disease, lung cancer with ongoing radiotherapy, blood or bone marrow cancer, ongoing chemo/immunotherapy, taking medications that weaken the immune system, sickle cell disease, cystic fibrosis or severe asthma/COPD, motor neuron disease, and patients asked to shield/self-isolate by the National Health Service.

Figure 1 – Author’s estimations from the UK COVID-19 symptoms tracker survey (April-December 2020, sample size: 16,771) and UK Governmental sources (UK Government, 2023): distribution of survey responses, new COVID-19 registered cases in the UK, and percentage of regions with a social mobility restriction measure (lockdown or Tier 3-4, as described in Section 2 of the manuscript) in place in 2020. Number of COVID-19 cases was rescaled (divided by ten). Percentage of regions with social mobility restrictions was rescaled (multiplied by ten) to improve visibility.


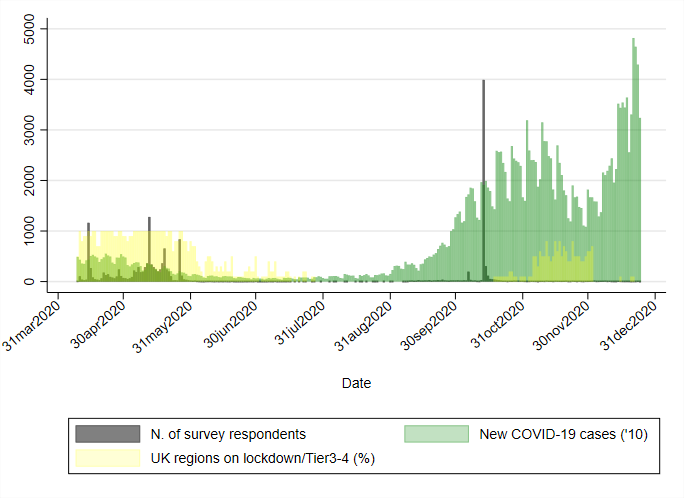


.

**References**

Spitzer, R. L., Kroenke, K., Williams, J. B. W. & Löwe, B. A Brief Measure for Assessing Generalized Anxiety Disorder: The GAD-7. Arch. Intern. Med. 166, 1092–1097 (2006).

Kroenke, K., Spitzer, R. L. & Williams, J. B. The PHQ-9: validity of a brief depression severity measure. J. Gen. Intern. Med. 16, 606–613 (2001).

UK Government (2023) Coronavirus tracker - Number of cases by specimen date. Coronavirus.Data.gov.uk Cases in United Kingdom. Last updated: March 16, 2023. Retrieved March 18, 2023, from https://coronavirus.data.gov.uk/details/cases.
